# Modification of Poly(Glycerol Adipate) with Tocopherol and Cholesterol Modulating Nanoparticle Self-Assemblies and Cellular Responses of Triple-Negative Breast Cancer Cells to SN-38 Delivery

**DOI:** 10.3390/pharmaceutics15082100

**Published:** 2023-08-08

**Authors:** Jiraphong Suksiriworapong, Chittin Achayawat, Phutthikom Juangrattanakamjorn, Vincenzo Taresco, Valentina Cuzzucoli Crucitti, Krisada Sakchaisri, Somnuk Bunsupa

**Affiliations:** 1 Department of Pharmacy, Faculty of Pharmacy, Mahidol University, Bangkok 10400, Thailand; 2 School of Chemistry, University of Nottingham, University Park, Nottingham NG7 2RD, UK; 3 Centre for Additive Manufacturing and Department of Chemical and Environmental Engineering, University of Nottingham, Nottingham NG7 2RD, UK; 4 Department of Pharmacology, Faculty of Pharmacy, Mahidol University, Bangkok 10400, Thailand; 5 Department of Pharmacognosy, Faculty of Pharmacy, Mahidol University, Bangkok 10400, Thailand

**Keywords:** poly(glycerol adipate), tocopherol, cholesterol, nanoparticle, SN-38, breast cancer

## Abstract

This study aimed to fabricate new variations of glycerol-based polyesters by grafting poly(glycerol adipate) (PGA) with hydrophobic bioactive moieties, tocopherol (TOC), and cholesterol (CHO). Their effects on nanoparticle (NP) formation, drug release, and cellular responses in cancer and normal cells were evaluated. CHO and TOC were successfully grafted onto PGA backbones with 30% and 50% mole grafting. Increasing the percentage of mole grafting in both molecules increased the glass transition temperature and water contact angle of the final polymers but decreased the critical micelle concentration of the formulated particles. PGA-TOC NPs reduced the proliferation of MDA-MB-231 cancer cells. However, they enhanced the proliferation of primary dermal fibroblasts within a specific concentration range. PGA-CHO NPs minimally affected the growth of cancer and normal cells. Both types of NPs did not affect apoptosis or the cell cycle of cancer cells. PGA-CHO and PGA-TOC NPs were able to entrap SN-38, a hydrophobic anticancer drug, with a particle size <200 nm. PGA-CHO NPs had a higher drug loading capacity and a greater drug release than PGA-TOC NPs. However, SN-38-loaded PGA-TOC NPs showed higher toxicity than SN-38 and SN-38-loaded PGA-CHO NPs due to the combined effects of antiproliferation and higher cellular uptake. Compared with SN-38, the drug-loaded NPs more profoundly induced sub-G1 in the cell cycle analysis and apoptosis of cancer cells in a similar pattern. Therefore, PGA-CHO and PGA-TOC polymers have potential applications as delivery systems for anticancer drugs.

## 1. Introduction

Pharmaceutical and biomedical applications have made substantial use of biodegradable polyester polymers because of their biocompatibility and biodegradability [1]. Particulate formulations of polyesters with encapsulated active pharmaceutical ingredients have attracted significant commercial interest and scientific challenges [1,2,3]. However, only a few chemical modifications can be applied to the polymer backbone of the most common polyesters, such as poly(lactide) or poly(caprolactone). These limitations restrict the possibility of manipulating the physicochemical properties of commercially available polyesters. Poly(glycerol adipate) (PGA) is classified as a biodegradable polyester due to its breakable ester bond connecting repeating units [4,5]. PGA and its variants have recently been introduced in the pharmaceutical and biomedical fields owing to their adaptability, self-assembling capability, and enzymatic degradability [4,5,6,7,8,9,10,11,12]. PGA contains free hydroxyl groups attached to each repeating unit that facilitate feasible modification or conjugation with drugs and ligands using mild and harmless reactions to the linker bonds.

Modification of PGA with a variety of pendant substituents, including fatty acids [8,10,13,14], N-acyl amino acids [15,16,17], non-steroidal anti-inflammatory drugs [18,19], methotrexate [20], and fluorescent dyes [15,21], can manipulate the physicochemical and mechanical properties, drug loading capacity, and controllability of drug release. These PGA modifications result in a grafted chain structure for the amphiphilic polymers. The nature of the side chain and the substitution level impact the structure and aggregation behavior of nanoparticles (NPs) [10]. The pendant side chains of the grafted polymers may repel more strongly than linear polymers, leaving less space between the side chains. This may give rise to unique characteristics in the grafted polymers. Compared to linear architectures, the more compact structure of the grafted polymers results in increased drug loading, reduced burst effects, enhanced self-assembling capability, and a lower critical aggregation concentration [14,22].

Different architectures of side chains and the drug structure influence the physicochemical properties of the grafted PGA particles and the degree of interaction and entrapment of the drug in the polymer matrix. For example, octanoyl- and stearoyl-grafted PGAs self-assemble into nanoparticles (NPs) and entrap dexamethasone phosphate [6,9]. The drug loading capacity depends on the hydrophobicity of the grafted PGAs, which is affected by the percentage of substitution, length of the acyl chain, and molecular weight [9]. In contrast, the maximum loading and slowest release of cytosine arabinoside, a water-soluble drug, were attained with unmodified PGA NPs rather than stearoyl-substituted PGA NPs [6]. Furthermore, in the case of ibuprofen and ketoprofen, only the ibuprofen sodium salt could be incorporated into the alkyl chain-substituted NPs. However, the ibuprofen and ketoprofen base forms were entrapped in the unmodified PGA NPs [23]. Besides particle formation and entrapment efficiency, different architecture and drug molecules affect drug release profiles from PGA NPs. PGA NPs are sensitive to pH and hydrolytic enzymes, as exemplified by PGA polymeric prodrugs. The PGA-drug conjugates of methotrexate, indomethacin, and mefenamic acid demonstrated enzymatic and acid-sensitive release of drugs depending on the percentage of drug conjugation and the NP environment [18,19,20]. A few studies have investigated the behavior of PGA NPs at the cellular level in cancer cells [5,24,25]. The uptake of unmodified PGA NPs by medulloblastoma cells depends on the dose of NPs and incubation time and proceeds through specific endocytic processes [5]. The uptake of NPs depends on the surface and size of the NPs in colorectal and brain cancer cell lines [24,25]. These findings showed a variety of applications of modified PGAs for drug delivery systems, primarily depending on the type and number of grafted moieties and entrapped drugs [26].

D-α-tocopherol (TOC) and cholesterol (CHO) have similar hydrophobic properties. Nevertheless, they have different structural components. TOC consists of a heterocyclic (chromane) ring with a hydroxyl group and a hydrophobic aliphatic linear side chain, whereas CHO contains four aliphatic cyclic rings (sterol) with a hydroxyl group and hydrophobic aliphatic linear chain. In biological aspects, TOC exerts differential anticancer activity against different cancer cell lines and shows no toxicity to normal cells [27,28], whereas CHO plays an important role in membrane fluidity and serves as a crucial structural element in mammalian cell membranes [29]. Both TOC and CHO, acting as hydrophobic anchors, have been successfully grafted onto polymer backbones, improving the amphiphilic nature of the corresponding polymers. Furthermore, they have demonstrated self-assembling properties to form micelles and NPs, as well as the entrapment ability of hydrophobic drug and deliverability of numerous anticancer drugs [30,31,32,33,34,35,36,37,38,39,40,41,42]. These modified NPs overcame the limited dissolution of problematic drugs [33,43], strengthened their stability in physiological fluids [35,40,44], controlled and sustained the release of drugs [32,38], and enhanced penetration and uptake into tumor cells [34,36,37].

**Figure 1:**
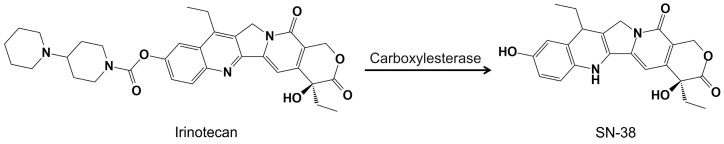
Bioactivation of irinotecan to SN-38, an active form, by carboxylesterase enzyme [46].

To our knowledge, hydrophobic modification of PGAs by CHO and TOC has never been reported. This is the first study comparing CHO- and TOC-grafted PGAs regarding particle formation, drug entrapment and release, and cellular responses in cancer and normal cells. While previous studies have explored various modifications of PGA using different grafted moieties, there has been a lack of investigation on the impact of non-linear hydrophobic grafted molecules on particle formation and behaviors of PGA-based systems for delivering hydrophobic anticancer drugs. The distinct structures of CHO and TOC may potentially influence their capability of drug entrapment, controllability of drug release, and delivery efficacy of anticancer drugs in different ways. In this study, CHO- and TOC-grafted PGA backbones were fabricated at nominal feeding 30% and 50% mole using a simple and mild coupling reaction. The structure of the grafted polymers was elucidated, and their properties were characterized in terms of thermal behavior, water contact angle (WCA), and critical micelle concentration (CMC). Particle formation and in vitro drug release of the developed particles were evaluated using SN-38, the active form of irinotecan (Figure 1), as a hydrophobic model. Understanding the cellular responses to modified PGAs is important because anticancer drugs must be delivered to cells or subcellular compartments for cancer treatment. However, there are no previous investigations into the effects of CHO- and TOC-grafted PGA NPs on normal and cancer cells. Therefore, we further investigated the effects of blank NPs on cell proliferation, cell cycle, and apoptosis of aggressive triple-negative MDA-MB-231 breast cancer cells [45] and compared them with primary dermal fibroblasts, adult (HDFa) cells. In addition, the efficacy of SN-38 delivery by CHO- and TOC-grafted NPs to MDA-MB-231 cells was evaluated regarding cytotoxicity, cellular uptake, cell cycle, and apoptosis of treated cells.

## 2. Materials and Methods

### 2.1. Materials

PGA was synthesized according to the previously reported protocol [14]. CHO hemisuccinate, 4-(dimethylamino)pyridine (DMAP), and TOC succinate were obtained from Tokyo Chemical Industry Co., Ltd., Chuo-ku, Tokyo, Japan. *N*,*N*’-dicyclohexylcarbodiimide (DCC), coumarin-6, and anhydrous tetrahydrofuran (THF) were purchased from Sigma-Aldrich, St. Louis, MO, USA. Phosphate buffered saline (PBS), fetal bovine serum (FBS), PrestoBlue^®^ cell viability reagent, and trypsin 0.25%-ethylenediaminetetraacetic acid (trypsin-EDTA) were purchased from Life Technologies Corporation, Eugene, OR, USA. Acetonitrile (ACN, high-performance liquid chromatography (HPLC) grade, Honeywell Burdick and Jackson, Muskegon, MI, USA), Dulbecco’s modified eagle medium (DMEM, high glucose, pyruvate, Invitrogen™, Thermo Fisher Scientific Inc., Waltham, MA, USA), Hoechst 33,342 (Invitrogen™, Thermo Fisher Scientific Inc., Waltham, MA, USA), and SN-38 (Suzhou Rovathin Foreign Trade Co., Ltd., Suzhou, China) were used as received. Sterile water for injection (General Hospital Products Public Co., Ltd., Pathumthani, Thailand) and deionized (DI) water were employed throughout this study. MDA-MB-231 breast cancer cell lines (ATCC number HTB-26^TM^, American Type Culture Collection, Manassas, VA, USA) and primary dermal fibroblasts, adult (HDFa, ATCC number PCS-201-012^TM^, American Type Culture Collection, Manassas, VA, USA) were cultured in DMEM medium supplemented with 10% FBS and 1% penicillin/streptomycin.

### 2.2. Syntheses of CHO- and TOC-Grafted PGA

CHO- and TOC-grafted PGA were synthesized through a carbodiimide-mediated coupling reaction as previously reported with some modification [47]. PGA (1 g, 4.95 mmol equivalent to OH groups) was dissolved in anhydrous THF (20 mL) with the subsequent additions of DMAP (4.95 mmol), CHO hemisuccinate, or TOC succinate (1.63 and 2.72 mmol equivalent to nominal 30% and 50% mole grafting), and DCC (1 mole equivalent to succinate of CHO and TOC). The reaction was conducted at room temperature for 24 h under a nitrogen atmosphere. The precipitate was removed by centrifugation, and the solvent in the supernatant was evaporated under reduced pressure. The crude polymer was washed with 0.2 M sodium hydroxide, 0.2 M hydrochloric acid, and water. The final purification was performed by dialysis of the polymer solution with methanol for 24 h. The resultant polymer was finally dried under reduced pressure at 45 °C for 24 h.

### 2.3. Polymer Characterization

#### 2.3.1. Structure Elucidation

The resultant polymers were characterized by proton nuclear magnetic resonance (^1^H NMR) and attenuated total reflectance infrared (ATR-IR) spectroscopies for structure elucidation. ^1^H NMR measurement was carried out by Bruker Avance NMR machine (Bruker Corporation, Rheinstetten, Germany) at 300 MHz and 25 °C in acetone-*d*_6_. ATR-IR spectroscopy was performed using Nicolet iS5 FTIR Spectrometer (Thermo Fisher Inc., Waltham, MA, USA) over the range of 4000–500 cm^−1^ at a resolution of 4 cm^−1^. The number-average molecular weight (M_n_) and molecular weight distribution (M_w_/M_n_) were measured by Agilent 1260 infinity GPC apparatus (Agilent Technologies, Inc., Cheadle, UK) equipped with light scattering and refractive index multi-detectors. Two Agilent PL-gel mixed D columns were used as a stationary phase for the sample elution in THF. The column oven was set at 40 °C, and the flow rate was 1 mL/min. M_n_ and M_w_/M_n_ were calculated from polymethylmethacrylate (PMMA) standards.

#### 2.3.2. Differential Scanning Calorimetry (DSC)

The DSC was performed to study the thermal behavior of the synthesized polymers. DSC was carried out by Q2000 differential scanning calorimeter (TA Instruments, Leatherhead, UK) under nitrogen flow at 50 mL/min. The polymers in aluminum pans were subjected to a heating/cooling cycle from −80 to 200 °C at a heating/cooling rate of 10 °C/min. Glass transitions were analyzed from the second heating.

#### 2.3.3. Water Contact Angle

The WCA measurement was conducted by KSV Cam 200 (KSV Instruments Ltd., Helsinki, Finland) [26]. The thin film of the synthesized polymers was prepared by dissolving them in acetone at a concentration of 3% w/w, dropping them on a glass microscope slide, and allowing them to dry. The tangent line was measured from a drop of distilled water dispensed by a flattened tip needle attached to a syringe onto the thin film of the polymer. The contact angle was analyzed in triplicate.

### 2.4. Nanoparticle Preparation

The NPs were prepared by a solvent-diffusion evaporation method [16]. In brief, the synthesized polymers (8 mg) were dissolved in 4 mL of acetone. The polymer solution was added drop by drop to 4 mL of distilled water under continuous stirring. While stirring, the solvent was evaporated in a fume hood at room temperature for 2 h. In the case of drug-loaded NPs, SN-38 was dissolved together with the polymer. The drug-to-polymer ratio was set at 0.2:10 by weight. The rest of the procedure was performed similarly as previously described.

### 2.5. Nanoparticle Characterization

The average hydrodynamic diameter, polydispersity index (PDI), and zeta potential (ZP) were measured by Zetasizer NanoZS (Malvern Instrument Ltd., Malvern, UK) equipped with a He-Ne laser at a wavelength of 633 nm, an angle of 173°, and 25 °C. The samples without dilution were measured in triplicate. The particle morphology was examined by a transmission electron microscope (TEM, JEOL JEM-1400, JEOL Ltd., Tokyo, Japan) on a formvar-coated grid and stained with 1% uranyl acetate.

As previously reported, the percentage of drug loading (%DL) was analyzed by direct and indirect methods [47,48]. For the direct method, the freshly prepared NPs (0.5 mL) were dried by freeze drying for 48 h using the lyophilizer (Crist Alpha 1–4 freeze dryer, SciQuip, Newtown, UK). The weight of lyophilized NPs was recorded for %DL calculation. The drug was extracted from the lyophilized samples by dissolving in THF and ACN and sonicating for 15 min. The sample was centrifuged at 12,000 rpm for 20 min, and the supernatant was collected and analyzed by HPLC. For the indirect method, 0.5 mL of the NPs as a dispersion form were filtered through a centrifugal filter device (MWCO 3 kDa, Ultracel^®^, Darmstadt, Germany) at 12,000 rpm for 20 min. The filtrate was collected and analyzed by HPLC. The entrapped drug in the NPs was computed by the difference product of drug amounts analyzed by direct and indirect methods. The %DL was then calculated according to Equation (1).

(1) $$\%DL=\frac{Analyzed\;amount\;of\;entrapped\;drug\;in\;the\;NPs}{Weight\;of\;lyophilized\;NPs}\times 100$$

For HPLC analysis of SN-38, an Agilent 1200 series HPLC machine (Agilent Technologies Inc., Santa Clara, CA, USA) was used to quantify the concentration of SN-38 in the samples. The drug was eluted through a reverse phase C18 HPLC column (Luna C18, 5 µm, 150 × 4.6 mm, Phenomenex Inc., Torrance, CA, USA) as a stationary phase by a mixture of ACN and phosphate buffer solution pH 3.1 at 25:75 *v/v* as a mobile phase at a flow rate of 1 mL/min. The eluent was detected by a diode array detector at a wavelength of 265 nm.

The CMC of blank NPs was analyzed using the pyrene fluorescence method [49,50]. Various concentrations of the NPs were incubated with 0.01 mM pyrene solution overnight. The fluorescence intensity of the resultant solutions was measured by a spectrofluorometer (Jasco FP-6200, Jasco International Co., Ltd. Tokyo, Japan) at excitation and emission wavelengths of 335 and 350–500 nm, respectively. The CMC values were calculated from the plot between the emission intensity ratio of 374 and 384 nm (I_374_/I_384_) and the concentrations of the NPs.

### 2.6. Drug Release Study

The release of SN-38 from the NPs was investigated using a dialysis method. The SN-38-loaded NPs and SN-38 solution, at an equivalent amount (20 µg) of SN-38, were filled into a dialysis bag (MWCO 1000 Da, Spectra/Por^®^, Spectrum Laboratories, Inc., Rancho Dominguez, CA, USA) and then immersed in 15 mL of PBS pH 7.4 containing 5% *v/v* dimethyl sulfoxide (DMSO) to maintain sink condition. The sample was shaken at 37 °C and 100 rpm for 168 h. At the predesignated times, 1 mL of release medium outside the dialysis bag was taken, and an equal volume of fresh release medium was immediately replenished. The release samples were immediately acidified with 50 µL of phosphoric acid to allow for the conversion of the drug molecule to the lactone form [48,51]. Finally, the sample was quantified by HPLC, as previously described. All experiments were performed in triplicate.

To further understand the release kinetics and mechanism of the developed NPs, the release profiles were fitted with the following kinetic equations: Zero order, first order, Higuchi’s, Korsmeyer–Peppas, and Hixon–Crowell models [52,53]. Correlation coefficient (R^2^), adjusted R^2^, and kinetic parameters were computed from the linear curve by regression analysis, and the Akaike information criterion (AIC) was utilized for model acceptance [54,55].

### 2.7. Cell Proliferation Study

The effect of the NPs on the proliferation of MDA-MB-231 breast cancer cells and HDFa primary dermal fibroblast cells was investigated using the resazurin-based assay [20,47]. The MDA-MB-231 and HDFa cells were seeded in a 96-well plate at a density of 5 × 10^3^ and 3 × 10^3^ cells/well, respectively. After 24 h, the cells were incubated with a series of NP concentrations (25–400 µg/mL in a fresh medium) at 37 °C in a 5% CO_2_ atmosphere for an additional 24, 48, and 72 h. At the designated times, PrestoBlue^®^ cell viability reagent was added and incubated for another 50 min. The CLARIOstar microplate reader (BMG LABTECH, Ortenberg, Germany) was utilized to measure the fluorescence intensity at an excitation wavelength of 535 nm and an emission wavelength of 590 nm. The relative cell growth was calculated using cell viability at each time point compared with the initial time point. All experiments were conducted in triplicate.

### 2.8. Cell Cycle Analysis

The cell cycle of MDA-MB-231 cells treated with SN-38, blank NPs, and SN-38-loaded NPs was evaluated by flow cytometry. The cells were seeded in a 6-well plate at 3 × 10^5^ cells/well density and incubated at 37 °C in a 5% CO_2_ atmosphere for 24 h. After that, the cells were treated with the samples for 24–72 h. After the predetermined time, the cell pellets were collected, stained with a cell cycle assay kit (Sigma-Aldrich Co. LLC., St. Louis, MO, USA), and analyzed by BD FACSVerse^TM^ flow cytometer (BD Biosciences, Franklin Lakes, NJ, USA). The experiments were performed in triplicate.

### 2.9. Cytotoxicity Study

The toxicity of SN-38-loaded NPs was evaluated in MDA-MB-231 cell lines using an MTT assay. The cells were seeded in a 96-well plate at 5 × 10^3^ cells/well density and incubated for 24 h prior to the treatment. Various concentrations of SN-38-loaded NPs and SN-38 solution were incubated with the cells for 48 h. Subsequently, 20 µL of MTT solution was added and incubated for another 2 h. Afterward, the medium was removed, and 100 µL of DMSO was added and incubated for 15 min. The absorbance at a wavelength of 570 nm was measured by the CLARIOstar microplate reader (BMG LABTECH, Ortenberg, Germany). The cell viability (%) was calculated, and an IC_50_ value was computed using GraphPad^®^ Prism version 7 software (GraphPad Software Inc., Boston, MA, USA).

### 2.10. Apoptosis Assay

The apoptotic study in MDA-MB-231 cells treated with the NPs was performed by flow cytometry. The cells were seeded in a 6-well plate at 3 × 10^5^ cells/well density and incubated at 37 °C under a 5% CO_2_ atmosphere for 24 h. The cells were then treated with the samples for 48 h. Thereafter, the cell pellets were collected, stained with a FITC Annexin V apoptosis detection kit I (BD Pharmingen™, BD Biosciences, Franklin Lakes, NJ, USA), and measured using BD FACSVerse^TM^ flow cytometer. The experiments were performed in triplicate.

### 2.11. Cellular Uptake Study

The uptake of coumarin-6-labeled NPs by MDA-MB-231 cells was investigated by flow cytometry). The cells were seeded in a 12-well plate at 3 × 10^5^ cells/well density and incubated at 37 °C under a 5% CO_2_ atmosphere for 24 h. Thereafter, the cells were exposed to 50 and 100 µg/mL coumarin-6-labeled NPs for 0.5, 1, and 2 h. Subsequently, the cells were washed twice with PBS, trypsinized with 0.25% trypsin/EDTA, and quenched with serum-containing media. The cells were then centrifuged, washed with PBS, and fixed with 4% paraformaldehyde for 30 min. The uptake was measured by a flow cytometer (BD FACSVerse^TM^). The results were expressed as the mean fluorescence intensity per event from three measurements.

For cellular uptake imaging, the cells were washed two times with PBS and fixed with 4% paraformaldehyde after 2 h of incubation with 100 µg/mL coumarin-6-labeled NPs. The Hoechst 33342 solution was added to the fixed cells and incubated for another 30 min prior to visualization by ZEISS LSM 900 confocal laser scanning microscope (Carl Zeiss AG, Oberkochen, Germany).

The coumarin-6-labeled NPs were prepared according to the previously described method in the NP preparation section. The characteristics of coumarin-6-labeled NPs were also evaluated. The coumarin-6-labeled NPs had comparable characteristics to the blank and drug-loaded NPs.

### 2.12. Statistical Analysis

The data are expressed as the mean ± standard deviation from at least three measurements. The t-test or One-way ANOVA with the Scheffe test applied post hoc for paired and multiple comparisons were performed to compare two or multiple groups, respectively. All analyses were determined using GraphPad^®^ Prism version 7 software, and differences were considered statistically significant at *p* < 0.05.

## 3. Results and Discussion

### 3.1. Polymer Characterization

#### 3.1.1. Structure Elucidation

**Figure 2:**
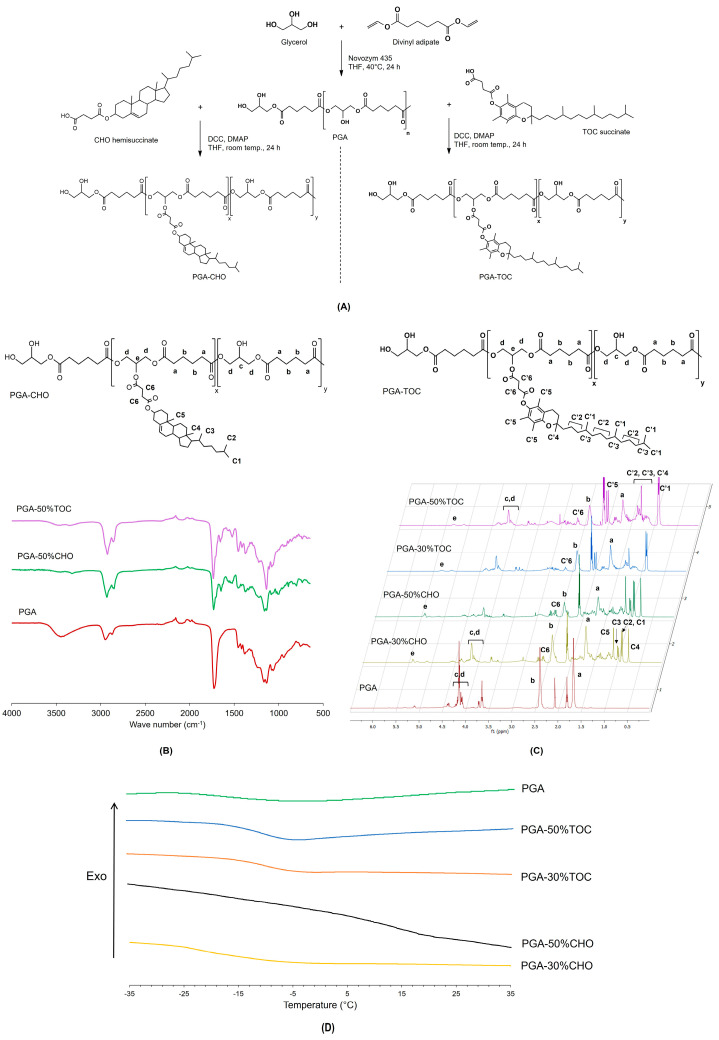
Synthesis scheme (**A**) exemplified ATR-IR spectra (**B**), ^1^H NMR spectra (**C**), and DSC thermograms (**D**, over the selected temperature range of –35 °C to 35 °C) of the synthesized PGA-CHO and PGA-TOC polymers.

As demonstrated in previous studies [6,9,11,12,23], using PGA without modification has limited drug delivery because of its low amphiphilic balance. The modification of PGAs with a certain number and type of substituted hydrophobic side chains has demonstrated a wide range of applications because of the modifiable physicochemical properties of NPs, enhanced drug loading capacity, and controllable drug release. Grafting different molecules onto the PGA backbone may modulate the physicochemical properties of the polymer and improve its potential for drug delivery applications. CHO and TOC, two hydrophobic endogenous compounds, have been extensively used to modify polymer properties and have been successfully applied in drug delivery systems [30,33,36]. Firstly, the grafting amount of CHO and TOC was predesigned to be nominally 10, 30, 50, and 80% mole grafting along the PGA backbone. The synthetic pathway is illustrated in Figure 2A. In the preliminary study, 10% and 80% mole grafting of CHO and TOC were successfully synthesized as elucidated by ^1^H NMR spectroscopy (Figure S1 in Supplementary Materials). However, these polymers could not entrap the model drug and form NPs. Therefore, only 30% and 50% mole grafting of CHO and TOC were further discussed in the following study and compared with PGA polymer.

The structure of the synthesized polymers was elucidated by ATR-IR and ^1^H NMR spectroscopies, as illustrated in Figure 2B and C, respectively. In the ATR-IR spectra (Figure 2B), the typical peaks of PGA at 3450, 2950–2870, 1725, 1130–1060, and 1075–1055 cm^−1^ were attributed to broad O-H stretching, symmetric and asymmetric C-H stretching of alkyl chains, C=O stretching, C-O-C stretching, and O-H bending of free primary and tertiary alcohols, respectively. These typical peaks of PGA were observed in the spectra of both series of grafted polymers. In addition, the C=O stretching peaks of the carbonyl ester shifted from 1725 to 1729 and 1732 cm^−1^, whereas new C=O stretching peaks appeared at 1702 and 1706 cm^−1^ in the spectra of PGA-CHO and PGA-TOC, respectively. A shift of the two O-C stretching bands of the ester bond to 1136 and 1158 cm^−1^ was observed in the spectrum of PGA-CHO. However, the C-O-C stretching peaks of PGA-TOC overlapped with the characteristic peaks of the TOC moiety. The C=C stretching peaks of CHO and TOC were detected at 1680–1652 cm^−1^. The broad O-H stretching peak of PGA at 3450 cm^−1^ were diminished after grafting. Other typical CHO and TOC peaks were observed in the spectra. Based on the ^1^H NMR spectra (Figure 2C), the methylene protons of adipic units of all grafted polymers appeared at 1.7 and 2.3 ppm, while those of glycerol units occurred at 3.8–4.3 ppm, comparable to those of the parent polymer. For the PGA-CHO polymers, the methyl protons of CHO at the C2, C4-C5, C3, and C1 positions were observed at 0.75, 0.88–0.90, 0.96–0.98, and 1.07–1.08 ppm, respectively. The methine proton peak at 5.31 ppm (peak e, Figure 2C) increased with the %mole of grafted CHO, suggesting an increase in 1,2,3-tri-substituted glycerol repeating units [14]. For the PGA-TOC polymers, the methyl protons of the aliphatic chain and phenyl ring of the TOC moiety were detected at 0.85–0.93 and 1.96–2.02 ppm, respectively, while the methylene proton peak occurred at 1.06–1.42 ppm. The methylene protons of the succinyl groups of CHO hemisuccinate and TOC succinate were observed at 2.59–2.67 ppm.

The percentage grafting of CHO and TOC was calculated from the ^1^H NMR spectra using methyl protons of the CHO and TOC moieties and methylene protons of the glycerol repeating units of PGA, as summarized in Table 1. The CHO and TOC grafting mole percentages were close to the theoretical values. As per previous studies on PGA, M_n_, and M_w_/M_n_ were reported to be approximately 11.6 × 10^3^ g/mol and 1.40, respectively. When grafted with CHO and TOC, the M_n_ values decreased slightly, whereas the distribution values increased (Table 1). A higher %grafting marginally increased the M_n_ values for CHO and TOC modifications. However, all the polymers showed broad dispersity values. Adding bulky functionalities as side chains changes the polymer hydrophobicity and the polymer architecture conformation (from linear to grafted/branched). The structural alteration led to a change in the solvated volume of the polymer and a consequently large variation compared to the linear PMMA standards used. The nature of PGA and its variant accounted for the differences from the adopted standards. The additional change in conformation due to grafting may account for the reduction in molecular weight. The increase in dispersity could be associated with the characteristic branch-chain architecture of the grafted polymers. These results show the successful modification of PGA polymers with CHO and TOC at 30% and 50% moles.

#### 3.1.2. Thermal Behavior

Introducing rigid, sterically hindered, aromatic moieties as side chains altered the amphiphilic balance and the thermal transitions, particularly the glass transition temperature (T_g_), thus, affecting the drug release behaviors. Bare PGA showed a single T_g_ at around –33 °C without the endothermic melting peak (Table 1). Grafting with CHO and TOC increased the T_g_ values of all polymers (Figure 2D). Despite the crystalline nature of CHO hemisuccinate and TOC succinate, the resulting polymers exhibited only one T_g_ step and no detectable melting peaks of CHO (T_m_ at 151 °C [56]) and TOC (T_m_ at 83 °C [57]) over the investigated temperature range (Figure S2). A higher percentage of CHO grafting increased the T_g_ values of the polymers. However, that of TOC grafting did not further increase their T_g_. All polymers retained a rubbery state at room temperature (25–30 °C). Our results confirmed that the CHO- and TOC-grafted PGA materials were amorphous.

#### 3.1.3. Water Contact Angle

The WCA was used to reflect the hydrophobicity of material surfaces. Increasing the number of substituted hydrophobic molecules decreased the number of free hydroxyl groups in PGA, resulting in more hydrophobic materials. Table 1 summarizes the WCA values of all studied polymers. PGA had the lowest WCA value suggesting the most hydrophilic property among all studied polymers. Compared with PGA, grafting CHO and TOC moieties onto the PGA backbones increased the WCA values linearly related to the % mole grafting (Figure S3). According to previous reports, TOC has a higher value of calculated partition coefficient than CHO (calculated log P:10.7 and 8.7, respectively [58,59]). PGA-50%TOC had a higher WCA value than PGA-50%CHO, suggesting a more hydrophobic character of PGA-TOC than PGA-CHO. However, a greater WCA value of PGA-30%CHO than that of PGA-30%TOC was observed, which was likely due to the higher % mole grafting of CHO (33.2%) than TOC (26.4%) when aiming for 30% mole functionalization.

These results indicated that the grafting of CHO and TOC affected the thermal behavior and hydrophobicity of PGA.

### 3.2. Particle Characterization

#### 3.2.1. Blank NPs

PGA-CHO and PGA-TOC NPs were prepared using a nanoprecipitation method. To investigate the effect of grafting CHO and TOC moieties on the characteristics of NPs, the blank PGA NPs were used for comparison. The CMC is frequently used to illustrate the stability and physical characteristics of micelles or nanosized aggregates. Nanosized particles with a low CMC value have an integrated structure resistant to dilution, which ensures their structural integrity upon dilution in the bloodstream [60]. CMC values of both series of NPs decreased with % mole grafting (Table 1). Increasing the number of grafted molecules increased the hydrophobicity of the polymers, positively affecting the amphiphilic balance and thus resulting in a lower CMC value. The CMC values of PGA-CHO and PGA-TOC NPs agreed with the number of grafted molecules. However, the CMC of the unmodified PGA NPs was considerably higher than that of the CHO- and TOC-grafted NPs. This result suggested that the grafting of CHO and TOC improved the self-assembly ability to form more stable and defined NPs than bare PGA.

**Figure 3:**
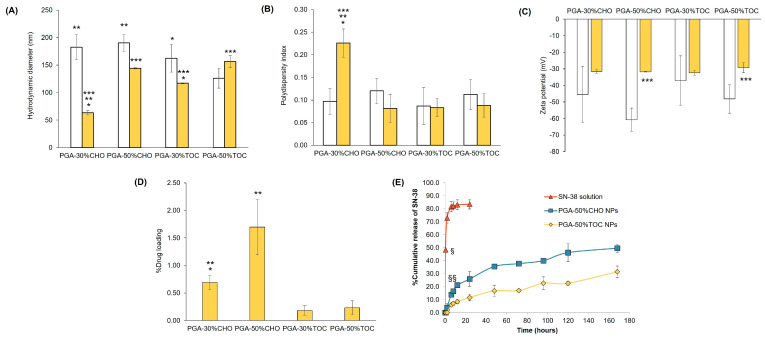
Hydrodynamic diameter (**A**, nm), polydispersity index (**B**, PDI), zeta potential (**C**, mV), and %drug loading (**D**) of blank and SN-38-loaded PGA-CHO and PGA-TOC NPs (n ≥ 3); release profiles (**E**) of SN-38 solution and SN-38-loaded PGA-CHO and PGA-TOC NPs (n = 3). * Significantly different comparing between 30% and 50% mole grafting; ** significantly different comparing between PGA-CHO and PGA-TOC at an equal mole grafting; *** significantly different comparing between blank and drug-loaded NPs; ^§^ significantly different comparing the SN-38 release of NPs and solution; and ^§§^ significantly different comparing the drug release of PGA-CHO and PGA-TOC NPs.

Regarding the NP properties, the empty PGA NPs possessed a particle size of 246 ± 2 nm, a PDI of 0.143 ± 0.22, and a ZP of –23.4 ± 0.8 mV. The hydrodynamic diameter and PDI of all blank grafted PGA NPs were smaller than 200 nm and narrower than 0.200 (Figure 3). The higher % grafting slightly enlarged the PGA-CHO NP diameter (183 ± 23 and 190 ± 15 nm for PGA-30%CHO and PGA-50%CHO NPs, respectively; *p* > 0.05). In addition, it significantly decreased PGA-TOC particle size (163 ± 25 and 126 ± 18 nm for PGA-30%TOC and PGA-50%TOC NPs, respectively; *p* < 0.05). Because PGA-50%TOC polymer exhibited the most hydrophobic property among other polymers, it may possess strong hydrophobic interaction inside the matrix core, resulting in the smallest particle size among other polymers. However, increasing the grafting percentages of CHO and TOC broadened the PDI values and lowered the ZP values of the blank NPs (*p* > 0.05). Furthermore, TOC conjugation significantly reduced the size of the NPs compared with the addition of CHO (*p* < 0.05). As the particle size depends on packing, more substituents may increase the lipophilicity and hydrophobic interactions within the particles [6]. In our system, the presence of TOC yielded the smallest particle diameter due to the coexistence of strong hydrophobic and π-π interactions of the alkyl chain and aromatic ring, respectively, in the core of the particles [61]. In contrast, although the CHO moieties had hydrophobic interactions in the particle core, a higher CHO substituent required more space. This could possibly be attributed to the increase in lipophilicity of grafted polymers, which led to an increased aggregation number of polymer molecules and the subsequent larger size of PGA-50%CHO particles [6]. Our study revealed that different types of grafted molecules, CHO and TOC, affected particle formation and characteristics differently.

**Figure 4:**
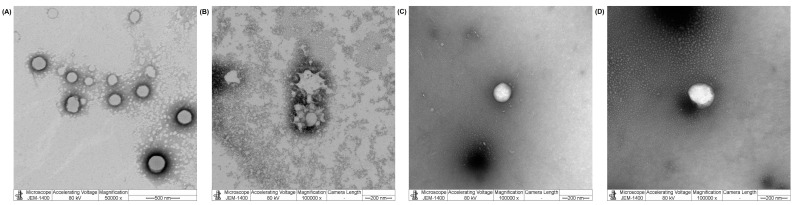
Examples of TEM images of blank PGA-50%CHO NPs (**A**), blank PGA-50%TOC NPs (**B**), SN-38-loaded PGA-50%CHO NPs (**C**), and SN-38-loaded PGA-50%TOC NPs (**D**).

TEM images of blank PGA-50%CHO and PGA-50%TOC are illustrated in Figure 4A and B, respectively. The blank PGA-50%CHO NPs had a rounded shape, while the blank PGA-50%TOC NPs had a more irregular shape than the PGA-50%CHO NPs. However, both NPs showed aggregation during the sample preparation.

#### 3.2.2. Drug-Loaded NPs

In this study, SN-38 was employed as a hydrophobic model drug to investigate the feasibility of PGA-CHO and PGA-TOC to deliver anticancer drugs. The NPs were prepared by the nanoprecipitation method without the addition of surfactants or stabilizers because this method governs the self-assembling property of amphiphilic polymers to form NPs and enables to evaluate the capacity of PGA-CHO and PGA-TOC NPs directly. Initially added SN-38 varied from 0.2:10 to 0.5:10 drug-to-polymer ratio in the preliminary study. As shown in Table S1, increasing the amount of SN-38 to 0.3:10 and 0.5:10 resulted in the formation of large aggregates upon preparation. After preparation, the large aggregates were removed by centrifugation, and the resulting dispersion was collected. The particle size, PDI, and ZP of drug-loaded NPs were comparable among all different drug-to-polymer ratios. Regarding loading capacity, %DL and %entrapment efficiency were highest when loading SN-38 at a 0.2:10 ratio except for PGA-30%TOC. The unchanged particle size and decreased loading capacity were possibly due to the limited capacity of the systems. Thus, this ratio was selected for further comparison study.

The characteristics of SN-38-loaded NPs are shown in Figure 3. After drug loading, both PGA-CHO NPs were considerably smaller than the unloaded particles (*p* < 0.05). The particle size of PGA-30%TOC NPs decreased, whereas that of PGA-50%TOC NPs increased, compared to the blank NPs (*p* < 0.05). Smaller sizes of SN-38-loaded PGA-CHO NPs and PGA-30%TOC NPs were associated with the interaction between the drug and matrix of the particle core, leading to a more compact and condensed core of the NPs. Meanwhile, the SN-38-loaded PGA-50%TOC NPs required more space for drug occupying with a subsequent larger size of NPs. All drug-loaded NPs had PDI values of <0.250, indicating a narrow size distribution. Most NPs had a narrower distribution after drug loading, except for PGA-30%CHO NPs, whose PDI increased significantly (*p* < 0.05). The zeta potentials of all drug-loaded NPs were less negative than those of the corresponding blank particles. Regarding loading capacity, the PGA-CHO NPs had a %DL of 0.69–1.70% and a %EE of 27.96–56.15% (Table S1), whereas the PGA-TOC NPs had considerably lower DL of 0.18–0.23% and EE of 7.31–9.68% (Table S1). A higher number of grafted molecules significantly enhanced the drug loading of the PGA-CHO NPs (*p* < 0.05), which was related to an increase in particle size. However, it minimally affected PGA-TOC NPs (*p* > 0.05). The higher drug loading of the PGA-CHO systems could be attributable to the higher compatibility between the drug and the grafted CHO moiety compared to the PGA-TOC systems. Since SN-38 is a hydrophobic analog of camptothecin, it could have had hydrophobic interactions with CHO or TOC. Thus, ATR-IR spectroscopy was employed to investigate the possible hydrophobic interactions between the drug and grafting molecules (CHO and TOC). Figure S4 shows changes in the peak pattern and frequency of C-H stretching and bending, phenolic C-O stretching, aromatic C=C stretching, and =C-H stretching of SN-38 and CHO succinate were observed, suggesting strong hydrophobic interactions between SN-38 and the CHO moiety of the PGA-CHO polymers. Similarly, changes in the peak pattern and frequency of SN-38 and TOC functional groups were detected; however, to a lesser extent than in the SN-38/CHO system, indicating weaker interactions between SN-38 and TOC moiety, thus limiting the drug loading capacity of the PGA-TOC systems. SN-38 may interrupt the interactions between polymer matrices in the PGA-50%TOC matrix core and account for the increased particle size after drug loading compared to the blank NPs. The hydrophobic interactions may involve van der Waals or π-π stacking between the drug and grafted moiety. TEM images of SN-38-loaded PGA-50%CHO and PGA-50%TOC (Figure 4C,D) showed that both drug-loaded NPs had a spherical shape. In the case of PGA-50%TOC NPs, incorporating SN-38 into the NPs resulted in a more spherical shape and fewer aggregates than the blank NPs. Based on these results, PGA-50%CHO and PGA-50%TOC NPs were chosen for further study of drug release.

The release profiles of SN-38 from PGA-50%CHO and PGA-50%TOC NPs compared with the drug solution are shown in Figure 3E. The release of SN-38 solution reached 80% within 6 h, suggesting that the dialysis bag did not delay the release of the drug. The release of SN-38 from PGA-50%CHO and PGA-50%TOC NPs showed sustained release over 168 h, with the total amount of drug release being 50% and 32%, respectively. The PGA-50%CHO NPs gave a significantly higher release of SN-38 than the PGA-50%TOC NPs after 6 h. Although the drug was more compatible with CHO, as evidenced in ATR-IR spectra (Figure S4), the higher drug release from PGA-50%CHO NPs may be attributable to the following factors. The first factor is that higher drug loading of the NPs elevates concentration gradient and driving force for drug diffusion from the NPs, and thus increases release rate and extent than the formulations having lower drug loading [62,63]. In addition, the polymer chain mobility of the particle core increased with the amount of encapsulated drugs [63] and was attributed to higher drug release. The second factor is the hydrophilicity of polymers. More hydrophilic polymers allow greater amounts of water to penetrate the particles and thus facilitate drug release from the NPs [62,64]. The last factor is the particle size. Smaller particles possess greater surface area, which causes more rapid drug release into the medium [62]. From our results, PGA-50%CHO NPs had approximately seven times greater %DL than PGA-50%TOC NPs, while the former had a slightly smaller size than the latter (144 ± 1 and 156 ± 11 nm, respectively). Moreover, PGA-50%CHO polymer had a lower WCA value (104 ± 1) than PGA-50%TOC polymer (113 ± 1), reflecting that PGA-50%CHO exhibited more hydrophilic properties than PGA-50%TOC. These differences in %DL, particle size, and WCA value possibly resulted in much more concentration gradient and chain mobility, larger surface area, and higher water penetration into the particles, respectively, and eventually led to higher drug release from PGA-50%CHO NPs as compared to PGA-50%TOC NPs. The release kinetics of SN-38 from both NPs were studied by fitting the release profiles to the following models: Zero order, first order, Higuchi’s, Korsmeyer–Peppas, and Hixson–Crowell models. The adjusted R^2^ and AIC were employed for a goodness-of-fit criterion based on corrected statistical errors and maximum likelihood [52,65]. The adjusted R^2^ closest to 1.0 and the lowest AIC value determined the best fitting of the model to the release profiles. From the fitting parameters shown in Table 2, the release profiles of PGA-50%CHO and PGA-50%TOC NPs fitted Korsmeyer–Peppas and Higuchi’s models, respectively. In the case of PGA-50%CHO NPs, the release exponent (n value in Table 2) was used for interpretation and found to be 0.3687. The release kinetics of spherical particles with a release exponent of 0.43 or less were considered to follow Fickian diffusion [52]. The drug release from PGA-50%TOC NPs was proportional to the square root of time and followed the Fickian diffusion mechanism. Therefore, the release kinetic fitting models suggest the Fickian diffusion mechanism of SN-38 released from both NPs, by which the drug diffusion or solvent transport rate was much greater than the polymeric chain relaxation. The release rate of SN-38-loaded PGA-50%CHO was higher than that of PGA-50%TOC NPs, as seen by the slopes of fitting kinetic models.

### 3.3. Effect on Cellular Responses

#### 3.3.1. Blank NPs

**Figure 5:**
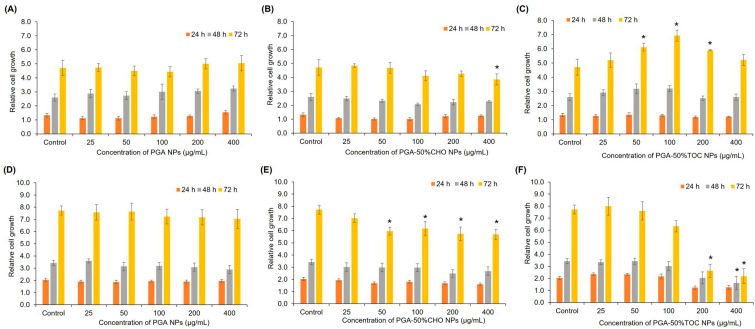
Proliferative profiles of PGA NPs (**A**,**D**), PGA-50%CHO NPs (**B**,**E**), and PGA-50%TOC NPs (**C**,**F**) in HDFa (**top row**) and MDA-MB-231 (**bottom row**) cells, n = 3. * Significantly different compared with the control group at an equal incubation time.

The effects of the blank NPs on the proliferation and cell cycle of normal and cancer cells were evaluated. This study used two cell lines: HDFa normal cells and MDA-MB-231 cancer cells. The cells were exposed to various concentrations of blank PGA-50%CHO and PGA-50%TOC NPs for 24, 48, and 72 h. Blank, unmodified PGA NPs were studied to evaluate whether the PGA backbone affected cell proliferation. As shown in Figure 5, none of the blank PGA NPs affected the proliferation of either cell line, even at the highest tested concentration (400 µg/mL), up to 72 h (*p* > 0.05), indicating that the PGA backbone did not affect the proliferation of normal and cancer cells (Figure 5A and D, respectively). In the case of PGA-50%CHO NPs, the NPs (50–400 µg/mL) slightly decreased the cell growth of MDA-MB-231 cells by approximately 20% compared to the control group (*p* < 0.05). However, they had a slight effect on HDFa cells. The PGA-50%TOC NPs dramatically decreased the proliferation of MDA-MB-231 cells in a dose-dependent manner. Prolonged exposure to PGA-50%TOC NPs resulted in delayed cell proliferation, especially at the 200–400 µg/mL concentration, by at least 60% at 72 h of incubation compared to the control (*p* < 0.05). In contrast, they had a positive effect on HDFa cells. The NPs over the 50–200 µg/mL concentration range enhanced the growth of HDFa normal cells compared to the control (*p* < 0.05). The different behaviors of PGA-50%TOC NPs in normal and cancer cells were likely due to TOC molecules attached to the nanoparticles. TOC and its derivatives have been reported to promote fibroblast proliferation over a 5–100 µM concentration range [66,67,68]. Thus, the enhanced growth of HDFa cells was possibly due to the promoting effect of TOC.

**Figure 6:**
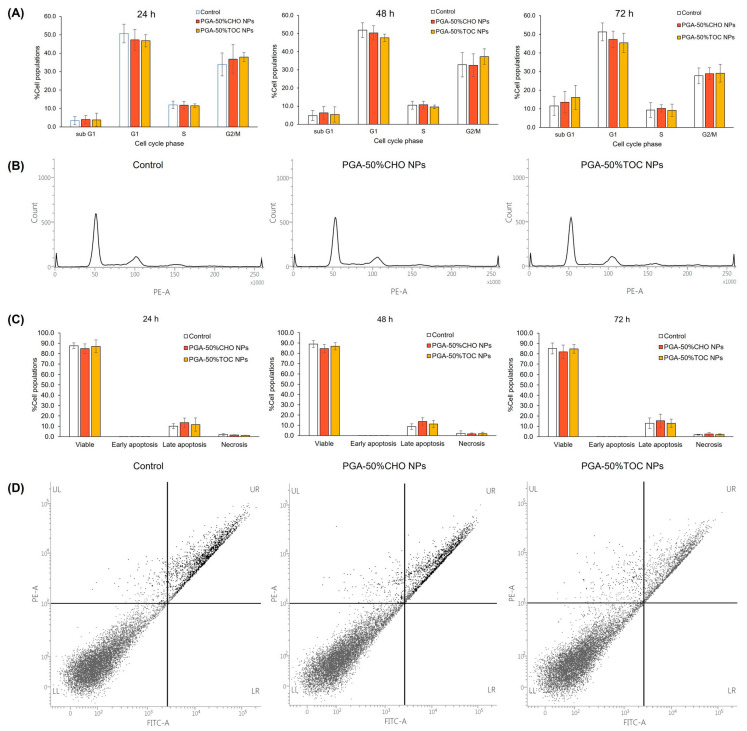
(**A**) Cell cycle profiles of MDA-MB-231 cells after treatment with the blank PGA-50%CHO and PGA-50%TOC NPs at 200 µg/mL for 24, 48, and 72 h compared to the untreated cells (n = 3) and (**B**) examples of cell cycle histograms at 72 h of incubation. (**C**) Apoptosis results of MDA-MB-231 cells after exposure to the blank PGA-50%CHO and PGA-50%TOC NPs at 200 µg/mL for 24, 48, and 72 h compared to the control (n = 3) and (**D**) examples of apoptosis quadratic diagram after treatment for 72 h.

According to the antiproliferative effects of PGA-50%CHO and PGA-50%TOC NPs on MDA-MB-231 cells, we further investigated the NP effect on the cell cycle. The cells were exposed to NPs for 24, 48, and 72 h because the antiproliferative effect was more pronounced when the exposure time was extended to 72 h. The cell cycle chromatograms and percentages of cell populations in all cellular phases are illustrated in Figure 6A,B. At 24 and 48 h of incubation with both NPs (Figure 6A), approximately 11 and 40% of the cells were in S and G2/M phases, respectively. When the exposure period was extended to 72 h, lower fractions of the S and G2/M phases were observed. However, the cell fraction in the sub G1 phase increased to approximately 13–16%. However, the cell populations and histogram patterns (Figure 6B) were comparable to those of untreated cells. We further evaluated the effect of bare PGA NPs on cell cycle distribution. As shown in Figure S5, PGA NPs did not significantly affect the cell cycle of MDA-MB-231 cells compared to the control. These results suggest that the NPs did not affect the populations of MDA-MB-231 cells at any of the cell cycle phases. The effect of blank NPs on the induction of apoptosis in MDA-MB-231 cells was investigated. The apoptosis profiles are illustrated in Figure 6C and D. After 24 h of treatment with PGA-50%CHO and PGA-50%TOC NPs, approximately 12–14% of the cells were in the late apoptotic phase, and small fractions were in the necrotic and early apoptotic phases. When extending the incubation period to 72 h, late apoptotic populations were comparable to the untreated group (15.38 ± 6.42%, and 12.97 ± 4.06%, respectively, *p* > 0.05). The results demonstrated that NPs promoted neither apoptosis nor necrosis in MDA-MB-231 cells compared to the control (*p* > 0.05). Dot-plot graphs (Figure 6D) showed similar patterns in the treated groups compared to the control group. Other studies have reported that TOC selectively impacts cell proliferation, differentiation, and apoptosis in different cell types [28]. However, not on normal cells, such as human peripheral monocytes or human skin fibroblasts [27]. In our study, the PGA-50%TOC NPs did not cause cell cycle arrest or apoptosis induction. We investigated the induction of apoptosis in MDA-MB-231 cells using TOC succinate, which was used to graft the backbone. At 55 µg/mL of TOC succinate, the representative concentration of TOC succinate in 200 µg/mL PGA-50%TOC NPs, it induced early and late apoptosis in MDA-MB-231 cells in a time-dependent manner. The total apoptotic populations of the cells increased from 19.33 ± 9.77% to 42.87 ± 14.17% and 50.37 ± 16.90% after 24, 48, and 72 h of incubation, respectively. Therefore, our study demonstrated no effect of PGA-50%TOC NPs on the apoptosis of MDA-MB-231 cells, although TOC molecules were attached to the PGA backbone. According to the results of studies on proliferation, cell cycle, and apoptosis, the PGA-50%CHO and PGA-50%TOC NPs affected the growth of MDA-MB-231 breast cancer cells with no interference in the cell cycle or apoptosis induction. However, the NPs did not affect the growth of normal cells. Therefore, they have potential applications in the delivery of drugs for the treatment of cancer.

#### 3.3.2. Drug-Loaded NPs

**Figure 7:**
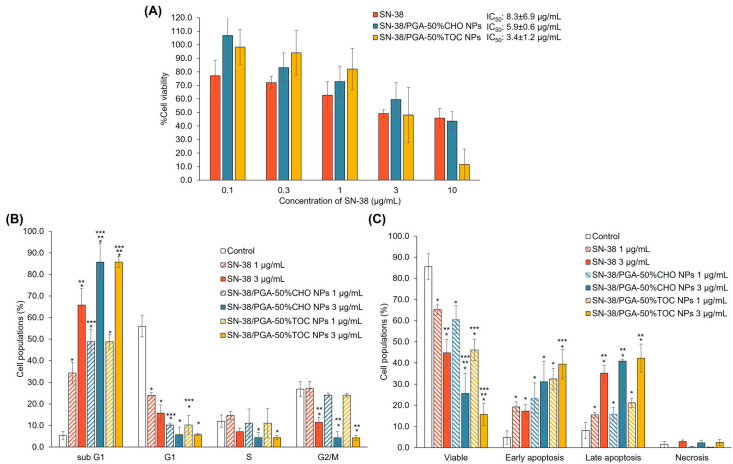
Cytotoxicity (**A**), cell cycle (**B**), and apoptotic (**C**) profiles of MDA-MB-231 cells after treatment with SN-38 (1 and 3 µg/mL) and SN-38-loaded PGA-50%CHO and PGA-50%TOC NPs equivalent to 1 and 3 µg/mL of SN-38 for 48 h (n = 3). * Significantly different compared to the control group; ** significantly different comparing between 1 and 3 µg/mL of SN-38 after treatment with the same sample; and *** significantly different compared to the cells treated with SN-38 solution at a similar concentration.

**Figure 8:**
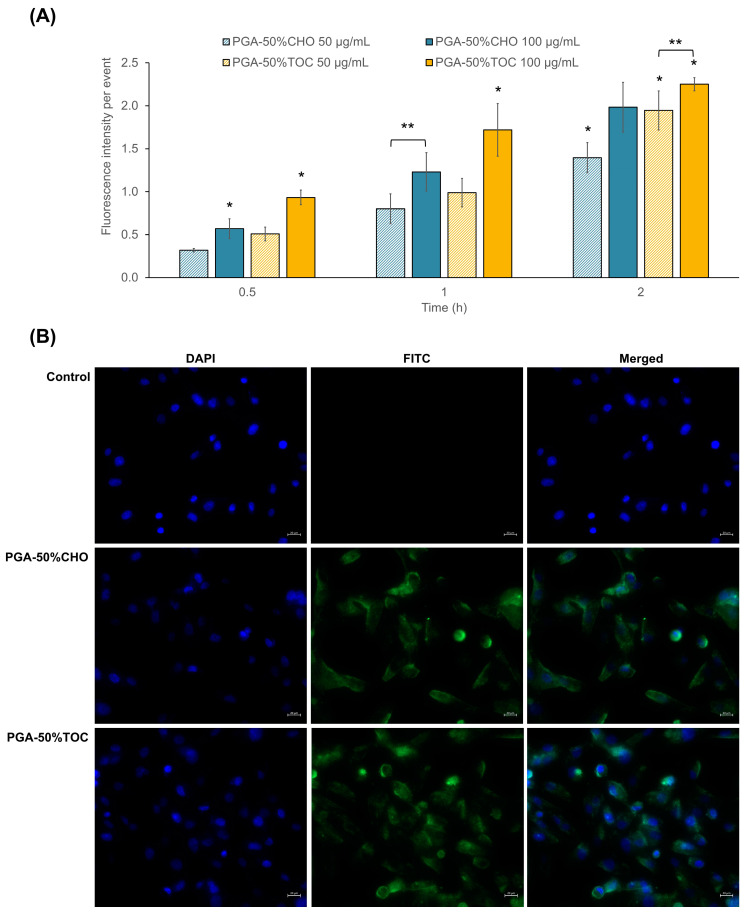
(**A**) Fluorescence intensity per events of MDA-MB-231 cells after exposure to coumarin-6 labeled PGA-50%CHO and PGA-50%TOC NPs at 50 and 100 µg/mL for 0.5, 1, and 2 h. An error bar indicates standard deviation (n = 3). * Significant difference when compared to the values at other time points; and ** significant difference when compared to the values at different concentrations. (**B**) Confocal laser scanning microscopic images of MDA-MB-231 cells after 2 h of incubation with 100 µg/mL coumarin-6-labeled PGA-50%CHO and PGA-50%TOC NPs compared to the untreated cells (control). DAPI and FITC channels illustrate the blue and green colors of Hoechst 33342-stained nuclei and coumarin-6-labeled NPs, respectively. The images were captured at a magnification of 40×. A scale bar indicates 20 µm length.

The toxicity of SN-38 and SN-38-loaded NPs was determined by the MTT assay in MDA-MB-231 cells after 48 h. As shown in Figure 7A, SN-38 exhibited an IC_50_ value of 8.3 ± 6.9 µg/mL. Among all samples, SN-38-loaded PGA-50%TOC and PGA-50%CHO NPs could potentiate the toxicity of SN-38 by 2.4 and 1.4 times, respectively. Although the drug release of SN-38-loaded PGA-50%CHO NPs was greater than that of PGA-50%TOC NPs, it was hypothesized that the lower toxicity of SN-38-loaded PGA-50%CHO NPs compared to PGA-50%TOC NPs was due to the combined effects of antiproliferative activity and cellular uptake of PGA-50%TOC NPs. As described above, PGA-50%TOC NPs suppressed the proliferation of MDA-MB-231 cells to a greater extent than PGA-50%CHO NPs. This effect may enhance the anticancer activity of SN-38 when loaded into PGA-50%TOC NPs. The cellular uptake of NPs may be another confounding factor that enhances the toxicity of SN-38-loaded NPs. Thus, we investigated the cellular uptake of coumarin-6-labeled NPs using flow cytometry. The uptake efficiency was quantified and expressed as a mean fluorescence intensity per event (Figure 8A). The results revealed that longer exposure times and higher NP concentrations increased the fluorescence intensity per event, suggesting that more NPs could enter the cells in a time- and concentration-dependent manner. The uptake of PGA-50%TOC NPs was tentatively higher than that of PGA-50%CHO NPs. Fluorescence images of the cellular uptake of NPs in MDA-MB-231 cells are illustrated in Figure 8B. PGA-50%TOC NPs showed higher intensities of green fluorescence around the blue-stained nuclei of the cells after 2 h of incubation than PGA-50%CHO NPs. It suggested that the NPs entered the cells and were located around the nucleus of the cells. These results could explain the lower toxicity of SN-38-loaded PGA-50%CHO NPs than PGA-50%TOC NPs, despite the higher drug release from PGA-50%CHO NPs.

We further evaluated the effect of the drug-loaded NPs on the cell cycle and apoptosis of MDA-MB-231 cells, as illustrated in Figure 7B and C, respectively. Based on the cell viability results showing that SN-38 at 1 and 3 µg/mL caused around 50% cell viability, the drug solutions at both concentrations were chosen for this study. The NPs containing SN-38 at a concentration equivalent to the drug solution were used. In Figure 7B, SN-38 at 1 and 3 µg/mL induced the cells in sub G1 by 34.24 ± 4.99% and 65.78 ± 7.75%, respectively. Nevertheless, SN-38 did not increase the cell populations in other cell cycle phases compared to the control. Both SN-38-loaded NPs similarly induced sub G1 to SN-38. The cell populations in the sub G1 phase after treatment with 1 and 3 µg/mL SN-38-loaded PGA-50%CHO NPs were 53.09 ± 5.84% and 85.44 ± 8.74%, respectively. Meanwhile, 48.71 ± 3.45% and 85.70 ± 2.55% of the cells were in the sub G1 phase when exposed to 1 and 3 µg/mL SN-38-loaded PGA-50%TOC NPs, respectively. When treated with a high dose of both NPs, the number of cells in the G1, S, and G2/M phases significantly decreased compared to the control group. In the apoptosis study (Figure 7C), SN-38 at 1 µg/mL induced early and late apoptosis by 19.26 ± 2.46% and 15.46 ± 1.23%. Higher concentrations of SN-38 predominantly induced a higher fraction of late apoptosis (35.13 ± 3.82%) and slightly affected a small fraction of necrosis (2.85 ± 0.77%). This result suggested apoptosis in MDA-MB-231 cells induced by SN-38 in a dose-dependent manner. The anticancer mechanism of SN-38 involves the inhibition of topoisomerase I [69]. SN-38 will form an inhibitory complex with topoisomerase I and DNA, resulting in single-stranded DNA breaks and apoptotic cell death through anti-apoptotic or pro-apoptotic protein cascades [70,71,72]. Our study demonstrated that SN-38 increased sub G1 cell populations with concomitant elevations in early and late apoptosis. When the cells were incubated with SN-38-loaded NPs, the number of cells in the early and late apoptotic phases increased dose-dependently. After treatment with 1 and 3 µg/mL SN-38-loaded PGA-50%CHO NPs, the early apoptotic cell populations were 23.36 ± 7.31% and 31.19 ± 9.64%, while those in late apoptosis were 15.80 ± 3.16% and 40.81 ± 0.73%, respectively. The increase in early and late apoptotic populations after treatment with SN-38-loaded PGA-50%CHO NPs was minimal compared to that in the drug-treated group (*p* > 0.05). In contrast, the exposure to SN-38-loaded PGA-50%TOC NPs gradually enhanced early and late apoptosis of the cells. At 1 µg/mL, SN-38-loaded PGA-50%TOC NPs led to 32.46 ± 4.97% and 21.18 ± 2.20% of the cells in early and late apoptotic phases, respectively. Increasing the NP doses to 3 µg/mL of SN-38 promoted the early and late apoptosis induction of the cells to 39.45 ± 6.92% and 42.27 ± 6.63%, respectively. As described above, the blank PGA-50%TOC and PGA-50%CHO NPs did not induce apoptosis of the cells or cell cycle arrest. The early and late apoptosis populations after treatment with both drug-loaded NPs were consistent with the cell populations in sub G1 phase. Therefore, the SN-38-loaded NPs promoted early and late apoptosis and sub G1 of MDA-MB-231 cells in a similar pattern but to a greater extent than the SN-38 solution. The combined results of cytotoxicity, cellular uptake, apoptosis, and cell cycle confirmed that PGA-50%TOC and PGA-50%CHO NPs could deliver SN-38 into the cells and enhance the anticancer effect of SN-38 toward MDA-MB-231 cells compared to the drug alone by inducing early and late apoptosis, which then caused cell death. This shows the promising potential for using these grafted polymers as delivery vehicles for anticancer drugs.

## 4. Conclusions

Our study revealed the successful modification of the PGA backbone by coupling CHO and TOC to adjust the physicochemical properties of the bare polyester backbone, including hydrophobicity, thermal behavior, and CMC. These modifications further altered the properties of the NPs and the delivery of SN-38 in MDA-MB-231 triple-negative breast cancer cells. However, these NPs did not affect the viability of the non-cancer cell lines used in this study. Therefore, the fabricated NPs show promising potential for the application of these grafted polymers as delivery systems for anticancer drugs. Nevertheless, the in vivo study may be required to demonstrate the safety and efficacy of the developed systems for future application, including the in vivo stability of the systems, biodistribution, antitumor efficacy, hemolytic effect, etc.

## Figures and Tables

**Table 1:** % Mole grafting, number-average molecular weight (M_n_), molecular weight distribution (M_w_/M_n_), glass transition temperature (T_g_), water contact angle (WCA), and critical micelle concentration (CMC) of synthesized polymers.

Polymer	% Mole Grafting	Mn (×10^3^ g/mol) [Mw/Mn]	T_g_ (°C)	WCA (°)	CMC (×10^−3^ g/L)
PGA	-	11.6 [1.4]	−33	55 ± 2	170 ^c^
PGA-30%CHO	33.2 ^a^	9.30 [3.2]	−17	96 ± 1	4.41
PGA-50%CHO	50.8 ^a^	10.5 [2.9]	8	104 ± 1	1.79
PGA-30%TOC	26.4 ^b^	9.70 [2.6]	−10	88 ± 1	4.52
PGA-50%TOC	50.4 ^b^	9.90 [3.4]	−11	113 ± 1	1.99

^a^ $\%\mathrm{Grafting}\;\mathrm{of}\;\mathrm{CHO}\;\mathrm{of}\;\text{PGA-CHO}=\frac{I_{0.75ppm}/3}{\left[I_{2.3ppm}-\left(\frac{I_{0.75ppm}\times 2}{3}\right)\right]/4}\times 100,$^b^$\;\%\mathrm{Grafting}\;\mathrm{of}\;\mathrm{TOC}\;\mathrm{of}\;\text{PGA-TOC}=\frac{I_{0.85-0.93ppm}/12}{I_{2.3ppm}/4}\times 100,$ where I_0_._75 ppm_ is an integral of methyl protons (3H) of the cholesteryl moiety at 0.75 ppm, I_2_._3 ppm_ is an integral of methylene protons (4H) of the glycerol repeating unit of PGA which, in the case of PGA-CHO, is overlapping with methylene protons (2H) of the CHO moiety at 2.3 ppm, and I_0_._85–0_._93 ppm_ is an integral of methyl protons (12H) of the TOC moiety at 0.85–0.93 ppm. ^c^ Value obtained from reference [12].

**Table 2:** Release kinetic model fitting parameters of SN-38 from PGA-50%CHO NPs and PGA-50%TOC NPs.

Model [Equation] ^a^	SN-38-Loaded PGA-50%CHO NPs				SN-38-Loaded PGA-50%TOC NPs			
	R^2^	Adjusted R^2^	AIC ^b^	Slope	R^2^	Adjusted R^2^	AIC ^b^	Slope
Zero order [F = kt]	0.895	0.895	−34.33	k = 0.0021	0.963	0.963	−50.84	k = 0.0015
First order [ln(1 − F) = −kt]	0.791	0.739	−26.18	k = 0.0071	0.870	0.838	−37.46	k = 0.0096
Higuchi’s [F = kt^1/2^]	0.723	0.723	−25.62	k = 0.0492	0.970	0.970	−52.50	k = 0.0233
Korsmeyer–Peppas [ln F = ln K + n ln t]	0.985	0.983	−50.80	K = 0.0793 n = 0.3665	0.985	0.969	−52.18	K = 0.0255 n = 0.4746
Hixson–Crowell [1 − (1 − F)^1/3^ = −kt]	0.829	0.818	−29.41	k = 0.0016	0.911	0.915	−43.27	k = 0.0017

^a^ F, t, and k mean fraction of drug released up to time t and release rate constant, respectively; K and n are a constant corresponding to geometric and structural characteristics and a release exponent determining the mechanism of drug release, ^b^ AIC stands for Akaike information criterion.

## Data Availability

The data presented in this study are available on request from the corresponding author.

## Supplementary Materials

The following supporting information can be downloaded at: https://www.mdpi.com/article/10.3390/pharmaceutics15082100/s1, Figure S1. ^1^H NMR spectra of PGA-10%CHO, PGA-80%CHO, PGA-10%TOC, and PGA-80%TOC polymers. The % mole grafting of these polymers, calculated based on ^1^H NMR spectra, were 19, 74, 3, and 51%, respectively. Figure S2. Examples of DSC thermograms of PGA-50%CHO and PGA-50%TOC polymers. Figure S3. Relationship of water contact angle and % mole grafting of PGA-CHO and PGA-TOC polymers. Figure S4. ATR-IR spectra of mixtures of SN-38 and (A) CHO succinate (CHOS) and (B) TOC succinate (TOCS) compared to SN-38 and their physical mixtures. Changes in pattern and frequency of peaks in the spectra of the mixtures compared with their individual and physical mixture spectra are indicated as red dashed lines. (A) SN-38/CHOS mixture: there were changes in frequency and intensity of peaks at 3452–3227, 2968, 2903–2863, 1600–1400, 1380–1250, and 900–750 cm^−1^ with a new peak at 1224 cm^−1^. (B) SN-38/TOCS mixture: There were changes in frequency and intensity of peaks at 3452–3227, 2968, 2903–2863, 1660–1630, and 1600–1400 cm^−1^. Figure S5. Cell cycle profiles of MDA-MB-231 cells after treatment with the blank PGA NPs at 200 µg/mL compared to the control (untreated cells) for 24, 48, and 72 h (n ≥ 3). Table S1. Characteristics of PGA-CHO and PGA-TOC NPs loaded with SN-38 at 0.2:10, 0.3:10, and 0.5:10 drug-to-polymer ratios
