# Supplementary material for: Modification of Poly(Glycerol Adipate) with Tocopherol and Cholesterol Modulating Nanoparticle Self-Assemblies and Cellular Responses of Triple-Negative Breast Cancer Cells to SN-38 Delivery
Source: Pharmaceutics. 2023 Aug 8;15(8):2100. doi: 10.3390/pharmaceutics15082100 (PMC10459774; doi:10.3390/pharmaceutics15082100)
Supplement: Supplementary file 1 [file pharmaceutics-15-02100-s001.zip › pharmaceutics-2528787-supplementary.pdf]

Supplementary Materials

Modification of Poly(Glycerol Adipate) with Tocopherol and Cholesterol Modulating Nanoparticle Self-assemblies and Cellular Responses of Triple-negative Breast Cancer Cells to SN-38 Delivery

Jiraphong Suksiriworapong [1,*], Chittin Achayawat [1], Phutthikom Juangrattanakamjorn [1], Vincenzo Taresco [2], Valentina Cuzzucoli Crucitti [3], Krisada Sakchaisri [4] and Somnuk Bunsupa [5]

[1] Department of Pharmacy, Faculty of Pharmacy, Mahidol University, Bangkok 10400, Thailand
[2] School of Chemistry, University of Nottingham, University Park, Nottingham NG7 2RD, UK
[3] Centre for Additive Manufacturing and Department of Chemical and Environmental Engineering, University of Nottingham, Nottingham NG7 2RD, UK
[4] Department of Pharmacology, Faculty of Pharmacy, Mahidol University, Bangkok 10400, Thailand
[5] Department of Pharmacognosy, Faculty of Pharmacy, Mahidol University, Bangkok 10400, Thailand
* Correspondence: jiraphong.suk@mahidol.edu

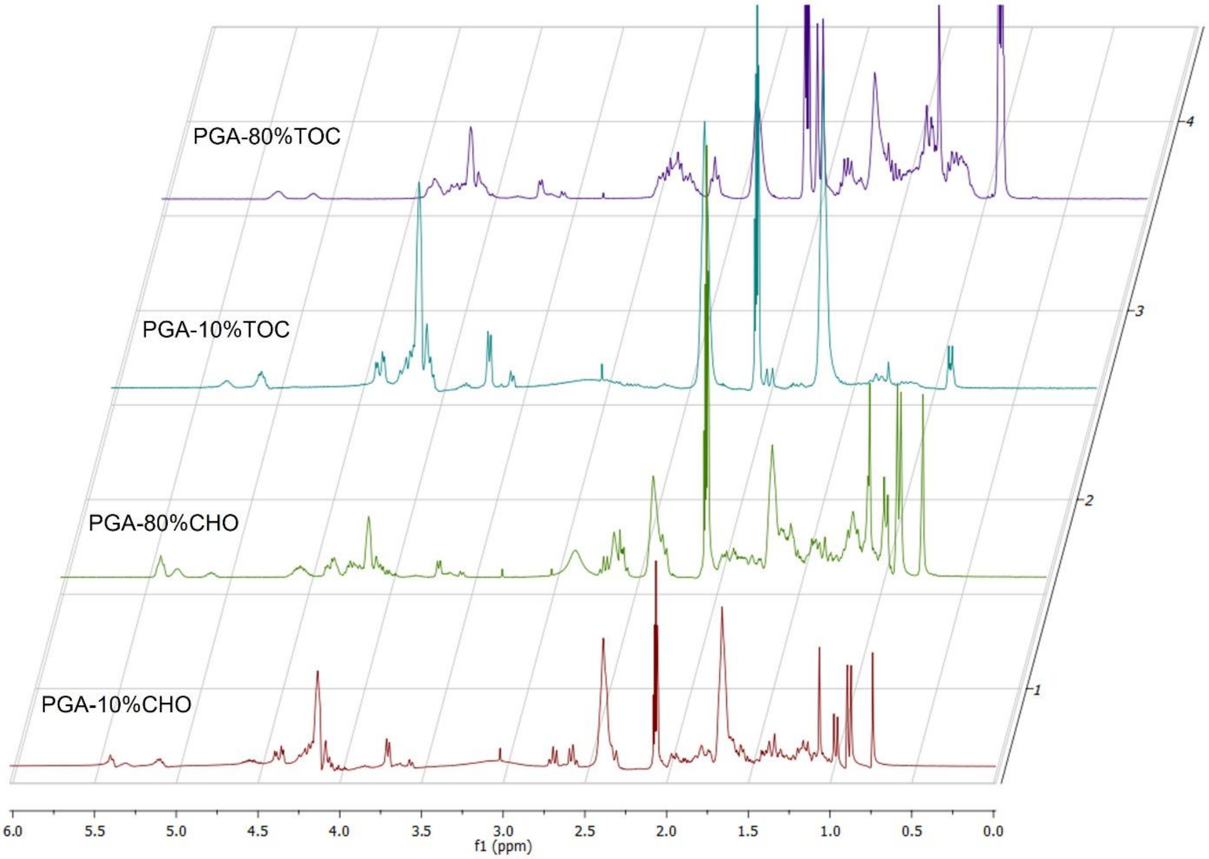

Figure S1. ^{1}H NMR spectra of PGA-10%CHO, PGA-80%CHO, PGA-10%TOC, and PGA-80%TOC polymers. The % mole grafting of these polymers, calculated based on ^{1}H NMR spectra, were 19, 74, 3, and 51 %, respectively.

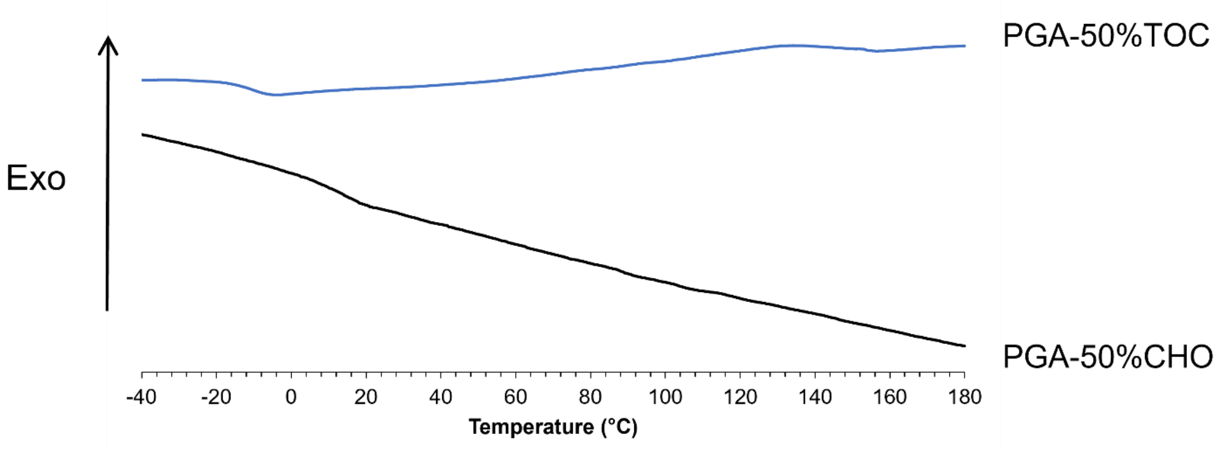

Figure S2. Examples of DSC thermograms of PGA-50%CHO and PGA-50%TOC polymers.

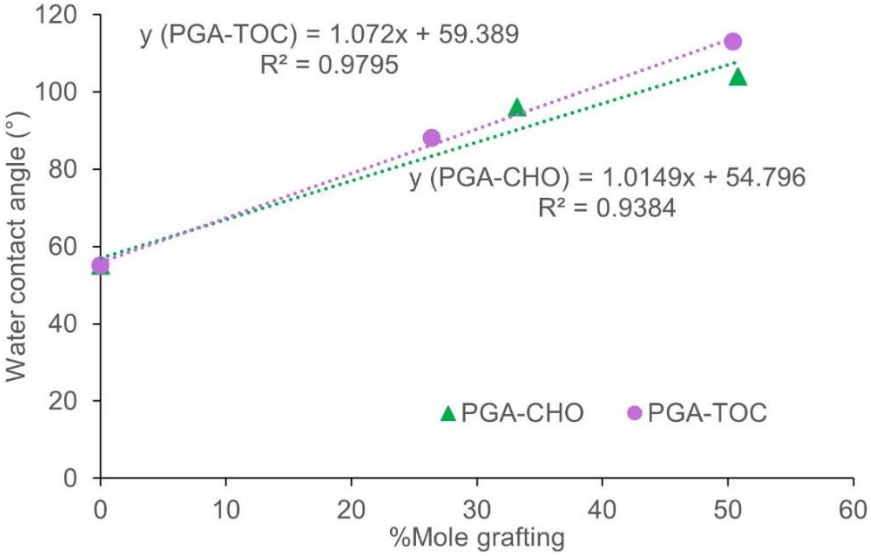

Figure S3. Relationship of water contact angle and % mole grafting of PGA-CHO and PGA-TOC polymers.

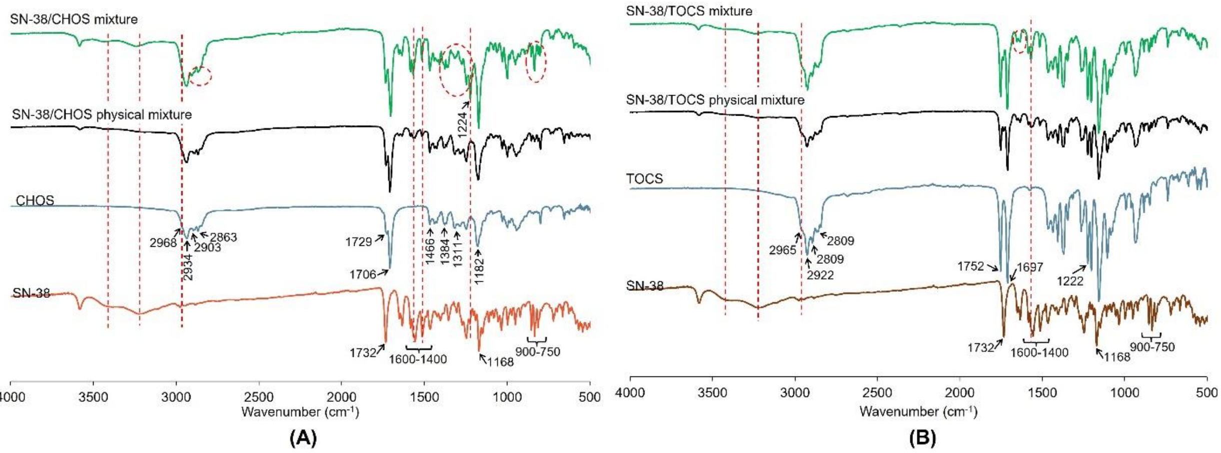

Figure S4. ATR-IR spectra of mixtures of SN-38 and (A) CHO succinate (CHOS) and (B) TOC succinate (TOCS) compared to SN-38 and their physical mixtures. Changes in pattern and frequency of peaks in the spectra of the mixtures compared with their individual and physical mixture spectra are indicated as red dashed lines. (A) SN-38/CHOS mixture: there were changes in frequency and intensity of peaks at 3452–3227, 2968, 2903–2863, 1600–1400, 1380-1250, and 900–750 cm^{-1} with a new peak at 1224 cm^{-1}. (B) SN-38/TOCS mixture: there were changes in frequency and intensity of peaks at 3452–3227, 2968, 2903–2863, 1660–1630, and 1600–1400 cm^{-1}.

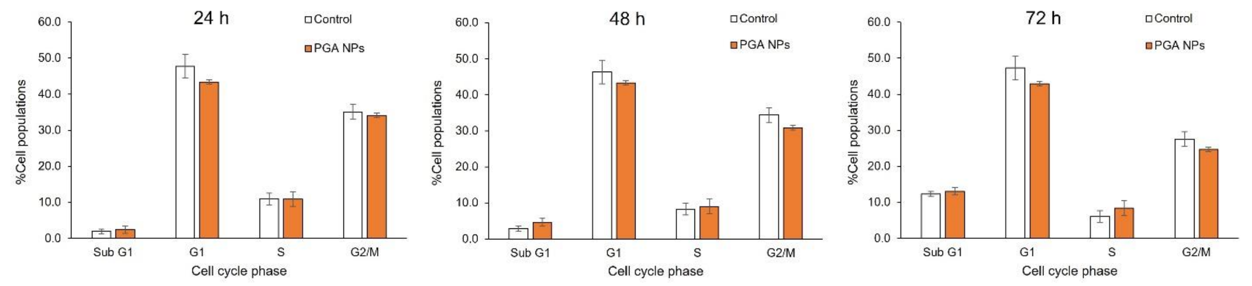

Figure S5. Cell cycle profiles of MDA-MB-231 cells after treatment with the blank PGA NPs at 200 μg/mL compared to the control (untreated cells) for 24, 48, and 72 h ($n \geq 3$).

Table S1. Characteristics of PGA-CHO and PGA-TOC NPs loaded with SN-38 at 0.2:10, 0.3:10, and 0.5:10 drug-to-polymer ratios

Polymer	Drug:Polymer ratio	Particle size (nm)	PDI*	ZP* (mV)	%DL*	%EE**
PGA-30%CHO	0.2:10	63±4	0.226±0.031	-31.5±1.2	0.69±0.13	27.96±4.34
	0.3:10	60±2	0.210±0.070	-30.0±6.2	0.28±0.07	6.79±0.62
	0.5:10	65±7	0.233±0.067	-33.2±4.6	0.35±0.02	5.99±0.71
PGA-50%CHO	0.2:10	144±1	0.082±0.031	-31.8±0.2	1.70±0.50	56.15±9.16
	0.3:10	142±3	0.093±0.019	-27.3±2.4	0.25±0.20	4.50±2.50
	0.5:10	147±3	0.089±0.024	-34.3±7.8	0.22±0.17	3.03±1.86
PGA-30%TOC	0.2:10	117±1	0.083±0.020	-32.4±1.4	0.18±0.09	7.31±3.66
	0.3:10	108±9	0.123±0.023	-31.5±3.8	0.51±0.17	7.45±1.86
	0.5:10	106±2	0.101±0.015	-31.6±1.8	0.40±0.07	5.40±0.31
PGA-50%TOC	0.2:10	156±11	0.088±0.026	-29.3±3.0	0.23±0.13	9.68±7.93
	0.3:10	140±4	0.087±0.024	-30.6±2.6	0.13±0.05	2.49±0.57
	0.5:10	144±2	0.089±0.024	-32.7±1.9	0.14±0.01	1.90±0.60

*PDI = polydispersity index, ZP = zeta potential, and %DL = %drug loading.

**%Entrapment efficiency $(\%EE) = \frac{Analyzed\ amount\ of entrapped\ drug\ in\ the\ NPs}{Jnitial\ amount\ of\ drug\ added} \times 100.$
